# Bioengineering potato plants to produce benzylglucosinolate for improved broad-spectrum pest and disease resistance

**DOI:** 10.1007/s11248-021-00255-w

**Published:** 2021-05-06

**Authors:** M. E. González-Romero, C. Rivera, K. Cancino, F. Geu-Flores, E. G. Cosio, M. Ghislain, B. A. Halkier

**Affiliations:** 1 Applied Biotechnology Laboratory, International Potato Centre, P.O. Box 1558, Lima, 12 Peru; 2 Department of Plant and Environmental Sciences, DynaMo Center, University of Copenhagen, Thorvaldsensvej 40, 1871 Frederiksberg, Denmark; 3 Department of Plant and Environmental Sciences, Copenhagen Plant Science Center & Section for Plant Biochemistry, University of Copenhagen, Thorvaldsensvej 40, Frederiksberg, Denmark; 4 Chemistry Section, Pontificia Universidad Católica del Perú, Av. Universitaria 1801, Lima, 15088 Peru; 5 Present Address: Universidad Nacional Agraria La Molina, Av. La Molina s/n, Lima, 12 Peru; 6 Present Address: Pathology Department, Instituto Nacional de Enfermedades Neoplásicas, Av. Angamos Este 2520, Lima, 15038 Peru

**Keywords:** Glucosinolates, Potato, Metabolic Pathway Engineering, Pest and Disease Resistance

## Abstract

In traditional, small-scale agriculture in the Andes, potatoes are frequently co-cultivated with the Andean edible tuber *Tropaeolum tuberosum,* commonly known as mashua, which is believed to exert a pest and disease protective role due to its content of the phenylalanine-derived benzylglucosinolate (BGLS). We bioengineered the production of BGLS in potato by consecutive generation of stable transgenic events with two polycistronic constructs encoding for expression of six BGLS biosynthetic genes from *Arabidopsis thaliana*. First, we integrated a polycistronic construct coding for the last three genes of the pathway (*SUR1, UGT74B1* and *SOT16*) into potato driven by the cauliflower mosaic virus 35S promoter. After identifying the single-insertion transgenic event with the highest transgene expression, we stacked a second polycistronic construct coding for the first three genes in the pathway (*CYP79A2, CYP83B1* and *GGP1*) driven by the leaf-specific promoter of the rubisco small subunit from chrysanthemum. We obtained transgenic events producing as high as 5.18 pmol BGLS/mg fresh weight compared to the non-transgenic potato plant producing undetectable levels of BGLS. Preliminary bioassays suggest a possible activity against *Phytophthora infestans*, causing the late blight disease and *Premnotrypes suturicallus*, referred to as the Andean potato weevil. However, we observed altered leaf morphology, abnormally thick and curlier leaves, reduced growth and tuber production in five out of ten selected transgenic events, which indicates that the expression of BGLS biosynthetic genes has an undesirable impact on the potato. Optimization of the expression of the BGLS biosynthetic pathway in potato is required to avoid alterations of plant development.

**Graphical abstract:**

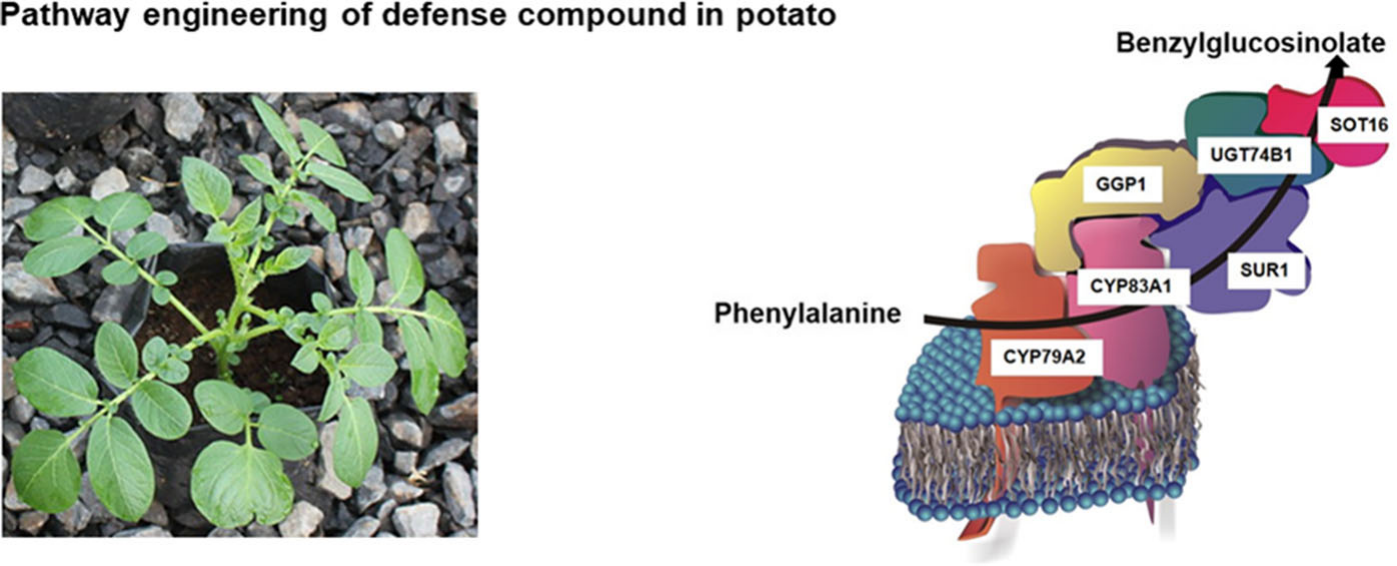

**Supplementary Information:**

The online version contains supplementary material available at 10.1007/s11248-021-00255-w.

## Introduction

Potato (*Solanum tuberosum*) is the world’s number one non-grain food commodity, with a global crop production that exceeds 368 million tons each year (FAO 2018). Potato production worldwide relies on intensive use of chemical pesticides to ensure stable yields. Many of the losses in potato production are related to the late blight disease caused by the oomycete *Phytophthora infestans* which is estimated to 35% in developing countries (Haverkort et al. 2016). The disease is primarily controlled by fungicides used up to 15 times per season and represent 50% of all pesticides in the Netherlands (Haverkort et al. 2016). In addition, insecticides control a wide range of insect pests including Colorado potato beetles, Andean potato weevils, potato tuber moths, leaf miner flies, white flies and aphids, which transmit potato leaf-roll virus and potato virus Y (Campos et al. 2019). Use of fungicides and insecticides is associated with costs of chemicals and machinery, energy and manual labor. This represents roughly 10% of the production costs (Haverkort el al. 2016). In addition, it puts farmers and their families at risk of intoxication due to inadequate use of protective equipment in small-scale and resource-poor production systems in developing countries (Damalas et al. 2011).

In traditional potato cultivation in the Andean highlands, knowledge and natural resources are exploited to reduce the impact of pests and pathogens. Here, the glucosinolate-producing *Tropaeolum tuberosum,* locally known as mashua, is used as a ‘barrier crop’ to protect neighboring potato plants from insects and pest attack (Grau et al. 2003). The biochemical basis for this ethnobotanical practice relies in the fact that mashua contains the defense-related, sulphur-rich compounds called glucosinolates (Johns et al. 1981; Ramallo et al. 2004). Being present as natural products in cabbage, broccoli and other less known Brassica vegetables such as mashua, glucosinolates are safe for human consumption and reported to function as cancer-prevention agents and flavor compounds. Glucosinolates and their hydrolytic enzymes, the myrosinases, are components of the glucosinolate-myrosinase system, also called the “mustard oil bomb”, which is characteristic of plants of the Brassicales order. Both components of the system are localized in different cell types in the plant tissue. Upon cellular disruption, the two components get into contact resulting in a variety of toxic compounds of which the major ones are isothiocyanates and nitriles (Halkier et al. 2006). The volatile isothiocyanates and other hydrolysis products liberated from mashua tissues upon chewing by insects are likely to protect the potato plants located in the middle of the field against a wide range of pests and diseases (Grau et al. 2003). Glucosinolates have been reported with a wide range of activities associated with pest and disease defense (Hopkins et al. 2009; Poveda et al. 2020). Benzylglucosinolate (BGLS) has been identified as one of the main glucosinolates accumulating in mashua (Johns et al. 1981; Ortega et al. 2006). This primed the idea to establish BGLS production within the potato plant itself. Engineering the production of the glucosinolate defense compounds into aerial parts of potato plants is expected to provide them with a novel defense system against insect herbivores and pathogens.

Metabolic pathway engineering presents several challenges such as concerted expression of the transgenes and phyto-toxicity of the intermediates or byproducts which may differ between the source species and the target species. One of earliest metabolic engineering project, the accumulation of β carotene in rice grain, had to optimize enzymatic activity of rate-limiting steps of the pathway to obtain enough pro-vitamin A content in the endosperm (Paine et al. 2005). In banana, optimization by testing different promoters was required to reach the desirable amount of pro-vitamin A content in the fruit (Paul et al. 2005). These optimization strategies included testing genes and promoters from different species to restrict the expression to specific organ or to development phases.

**Fig. 1:**
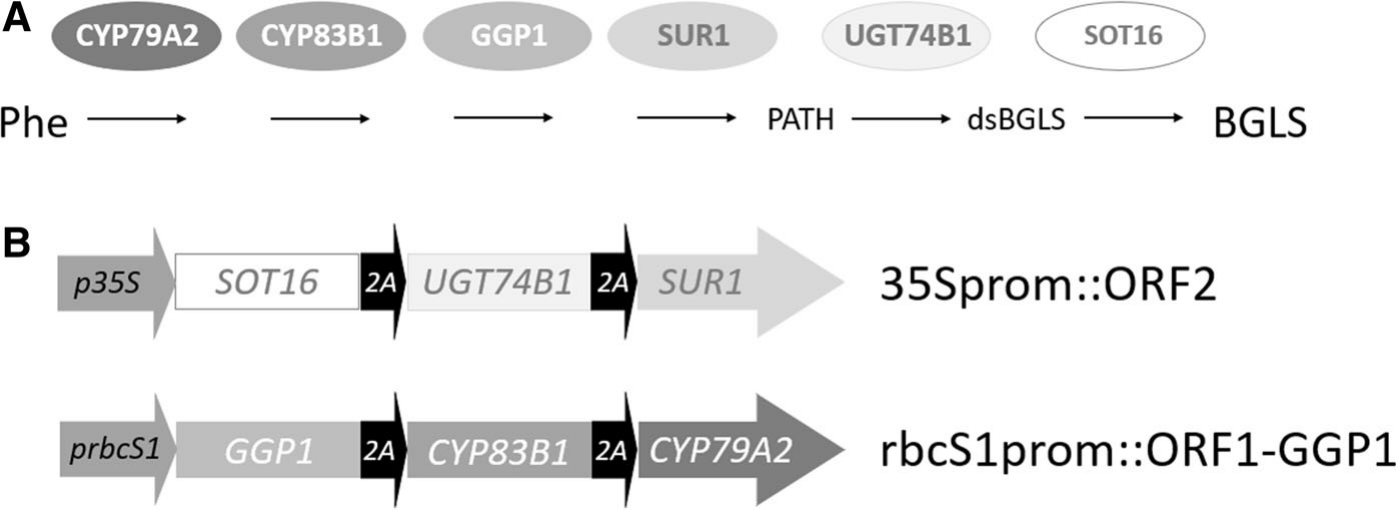
Strategy for engineering benzylglucosinolate (BGLS) production into potato. A. Genes in the BGLS pathway in *Arabidopsis thaliana* introduced into potato. Selected intermediates of the *ORF1-GGP1* transgene are designated. B. Schematic view of the two multigene transgenes generated for production of BGLS in potato. *ORF2* is driven by 35S promoter (p35S) whereas *ORF1-GGP1* is driven by rubisco S1 promoter (prbcS1). The 2A sequences added to the two first genes in replacement of stop codon in each of the transgenes enable that the polycistronic mRNA is translated into three individual polypeptides. Abbreviations: Phe—phenylalanine, PATH—phenylacetothiohydroximic acid, dsBGLS—desulfobenzylglucosinolate, GGP1—γ-glutamyl peptidase, SUR1—C–S lyase, UGT74B1—UDPG glucosyltransferase, SOT16—sulfotransferase

The production of the phenylalanine-derived BGLS has been engineered into non-cruciferous tobacco plants using six genes from *Arabidopsis thaliana* (Geu-Flores et al. 2009a, b; Møldrup et al. 2012). The first three genes convert phenylalanine into S-alkylthiohydroximate using two cytochromes P450 (CYP79A2 and CYP83B1) and γ-glutamyl peptidase 1 (GGP1). The last three genes including a C-S lyase (SUR1), a glucosyltransferase (UGT74B1) and a sulfotransferase (SOT16) result in formation of BGLS (Fig. 1a).

Several strategies need to be considered when engineering the six genes required for BGLS production in potato. First, the six genes of the BGLS pathways were from the cruciferous plant *A. thaliana* because those of *T. tuberosum* mashua were not available. Secondly, stable gene expression of all six genes of the pathway can be achieved by introducing all transgenes into the same T-DNA and selecting the transgenic event where gene expression is optimal. This would require using six different promoters with the same expression pattern to reduce homology-dependent gene silencing (Meyer et al. 1996). An alternative to using different promoters is the use of the self-procession polypeptide using the 2A oligonucleotide linking coding sequences of the transgenes under the control of one single promoter (de Felipe et al. 2006). The individual proteins derived from such a polycistronic construct will be tagged with the ~ 18 amino acid 2A, except for the terminal protein. Accordingly, this may influence the order of the genes in the polycistronic construct. Previously, the strategy combining three coding sequences in two transgene constructs was proven successful in engineering BGLS production in tobacco (Geu-Flores et al. 2009b; Møldrup et al. 2012). Thirdly, the accumulation of toxic metabolites as intermediate products from pathway engineering or byproducts from pleiotropic activities is a concern when engineering multi-gene pathways (Morant et al. 2007). Therefore, the introduction of the genes ‘backwards’, i.e. with the last genes first, will prevent the accumulation of potentially toxic intermediates and has been a successful for BGLS production in tobacco (Geu-Flores et al. 2009b; Møldrup et al. 2012).

The production of the defensive BGLS hydrolysis products in potato to repel pests or inhibit pathogens such as *Phytophthora infestans* must take place in the leaves during the whole growing period of the plant. Promoters targeting potato leaf expression exists and can drive expression of transgenes during plant development at high level (Miroshnichenko et al. 2020). Among these the CaMV 35S is one of the most popular promoter used in potato driving constitutive expression and is therefore suitable for expressing the last steps of the BGLS pathway creating a strong flux for pathway intermediates towards the end product BGLS. Since the tubers are not targeted to avoid giving them a glucosinolate-like flavor, a light-dependent expression of the first three steps would restrict BGLS to the leaves. The ribulose-1,5-bisphosphate carboxylase small-subunit gene family of chrysanthemum provides such expression pattern in potato (Outchkourov et al. 2003). Hence a tissue-specific restriction of the first three genes followed by a non-organ specific flux of intermediates of the glucosinolate biosynthetic pathway is proposed as the strategy to minimize the accumulation of toxic intermediates.

Based on the use of the cruciferous crop mashua in potato traditional cultivation to control insect pests, we propose that BGLS may have a direct effect as repellant to insect herbivores and growth inhibitor of pathogens of the potato. Here, we report the stable integration of six genes in the BGLS pathway into the potato genome and the production of BGLS in transgenic potato plants. This is an important first step towards introducing the two-component glucosinolate-myrosinase system into potato to improve pest and disease resistance.

## Results

### Identification of transgenic potato events with the ORF2 transgene

The polycistronic construct 35Sprom::ORF2 consisting of the genes coding for the last three enzymes of the BGLS pathway in *Arabidopsis thaliana* (SOT16, UGT74B1 and SUR1) and the *nptII* selectable marker gene were introduced into potato by *Agrobacterium*-mediated transformation using a selection for transgenic events through their resistance to kanamycin (Fig. 1b). The 35Sprom::ORF2 construct was integrated first to avoid the production of toxic intermediates that could poison the young regenerants. Organogenic calli formed starting in the third week after infection and plantlets were regenerated from the callus four weeks later. Within a total of 234 regenerants, we used PCR to identify 27 putative transgenic events. We analyzed the expression of the *ORF2* transgene by measuring mRNA levels using primers in the *SUR1* coding sequence (Fig. S1). The *ORF2* transgene expression ranged between 0.5 and 4500 compared to the expression of the housekeeping *cox1* gene. The transgenic event with the highest expression was event 85, featuring three-fold higher expression levels than for the event with the second highest expression. Almost two-thirds of the transgenic events had much lower level of *ORF2* transgene expression than the top three. Southern blot analysis was conducted on the 27 PCR-positive transgenic events to evaluate transgene insertion number, as single-insertion events are easier to characterize molecularly for subsequent product development. Transgenic event 85 had a single insertion of the *nptII* gene linked to the *ORF2* transgene (Fig. S2A). Ten additional transgenic events were found to have a single transgene insertion (data not shown).

### Analysis of UGT74B1 and SOT16 activity in ORF2 transgenic plants

**Fig. 2:**
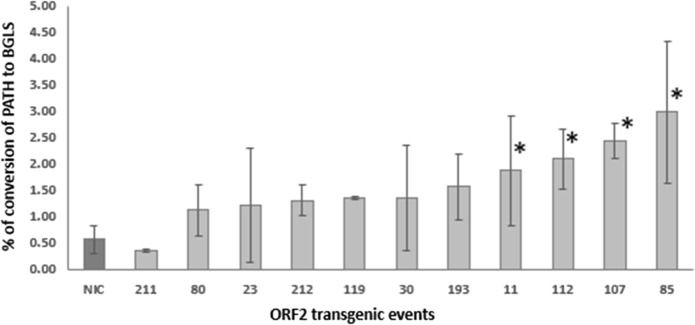
BGLS production upon in vivo feeding of PATH to leaves of untransformed potato variety Désirée (NIC) or transgenic events carrying the *ORF2* transgene, as analyzed by HPLC. Bar diagrams show the conversion percentage of PATH into BGLS in the 11 transgenic events and in the untransformed potato variety Désirée (NIC). Error bars represent standard deviation, n = 6. Asterisks indicate significant differences with the untransformed potato variety determined using Dunnett’s test (*P* < 0.05)

The eleven transgenic events containing a single *ORF2* transgene were analyzed for functional expression of the UGT74 glucosyl- and SOT16 sulfotransferase in vivo by feeding leaves with pathway intermediate phenylacetothiohydroximic acid (PATH) and measuring conversion into BGLS (Fig. 1a). HPLC analysis of BGLS levels in transgenic leaves fed with PATH showed a statistically significant difference between four transgenic events (11, 112, 105, 85 with conversion of 1.87%, 2.09%, 2.43%, 2.98% respectively) and the near-isogenic control (NIC), which showed a background conversion of 0.56% (*P* < 0.05 on a Dunnett's tests) (Fig. 2). Among these four transgenic events, event 85 displayed the highest conversion with an average that was approximately 6 times higher than for the wildtype control. This event 85 was also the one with the highest *ORF2* transgene expression (Fig. S1).

### Generation of BGLS-producing potato events

**Fig. 3:**
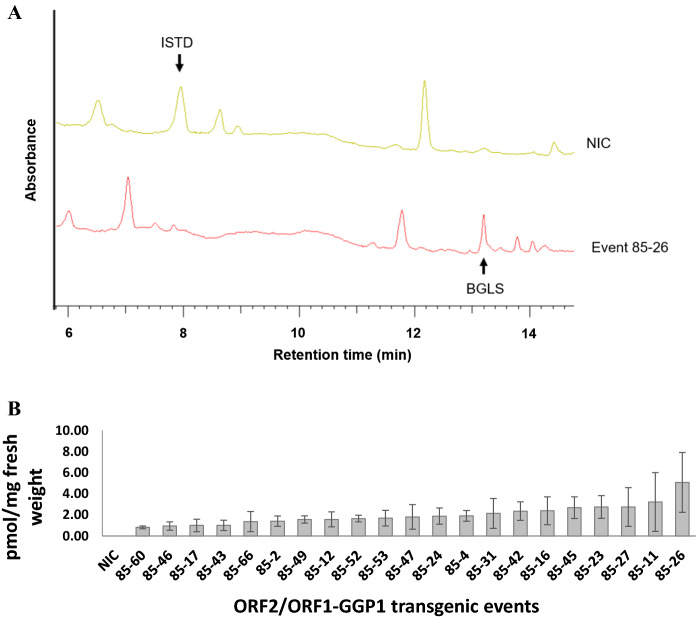
BGLS production in ORF2/ORF1-GGP1 transgenic plants. A. HPLC–UV chromatograms of extracts from an untransformed potato variety Désirée (NIC) compared to a selected transgenic event, event 26, carrying both the *ORF2* and *ORF1-GGP1* transgenes (event 26). The peaks of BGLS of the internal standard (sinigrin ISTD) are marked by arrows. B. BGLS production of in vitro*-*grown, five-week-old potato plants carrying both the *ORF2* and *ORF1-GGP1* transgenes, as quantified by HPLC. Error bars represent standard deviation, n = 6

Transgenic event 85 that displayed both the highest level of *ORF2* transgene expression and the highest level of PATH-to-BGLS conversion as well as with a single insertion of the *ORF2* transgene, was selected for retransformation with the rbcS1prom::ORF1-GGP1 construct with the *bar* selectable marker gene (Fig. 1b). Amongst 57 regenerants selected as resistant to phosphinothricin, we identified 55 transgenic events by PCR analysis. Methanol extracts from 50 out of 55 PCR positive transgenic events were analyzed for BGLS accumulation using HPLC. The remaining five events were not analyzed due to their abnormal phenotypes, which included poor growth characterized by a bushy aspect, a height of 10 to 15 cm and no leaves instead of 36 cm for the NIC plants. Accumulation of BGLS was detected in leaves of five-week-old plantlets in 21 of the transgenic events whereas no BGLS was detected in the NIC as expected (Fig. 3a). Transgenic event 85–26 showed the highest level of BGLS, averaging 5.18 pmol/g fresh weight. The accumulation of BGLS in this transgenic event was twice as high as that of the event with the second highest BGLS accumulation whereas the lowest BGLS detected was at 0.81 pmol/g fresh weight for the transgenic event 85–60 (Fig. 3b). Ten BGLS-producing transgenic events, including 85–26 with the highest BGLS accumulation, were analyzed for insertion number in relation to the *ORF1-GGP1* transgene. Only transgenic event 85–26 was found to have a single insertion of the *bar* gene (Fig. S2B).

### Effects of BGLS pathway expression on plant growth

**Fig. 4:**
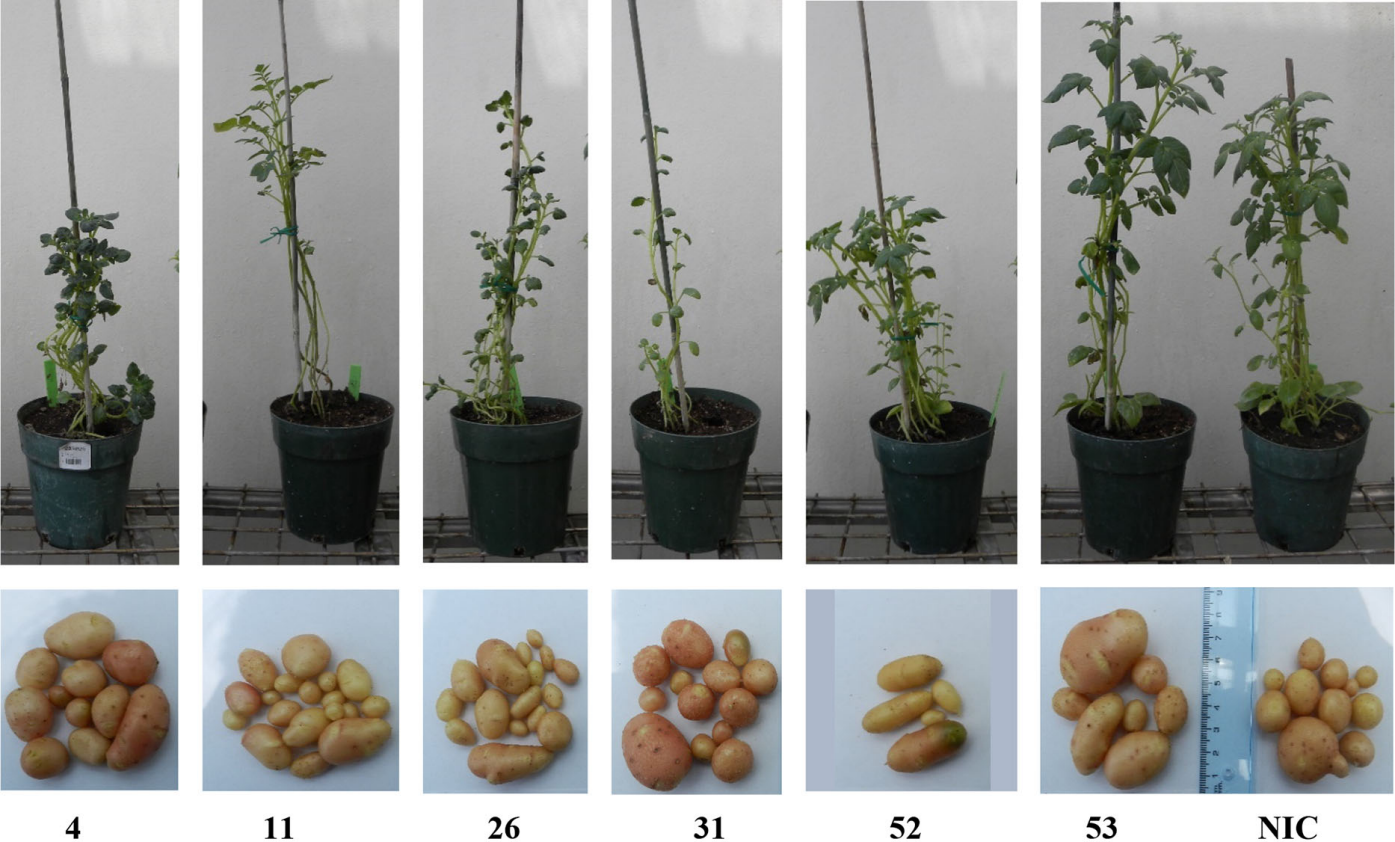
Morphology of six transgenic BGLS-producing potato transgenic events (4, 11, 26, 31, 52 and 53) in comparison to untransformed variety Désirée (NIC). Upper row: two-month-old plants; lower row: tubers

The growth of the ten transgenic events in the greenhouse was observed and their morphology evaluated. Four transgenic events (85–16, 85–23, 85–24 and 85–49) presented abnormally thick and curlier leaves and did not produce any tubers. The other six transgenic events (85–4, 85–11, 85–26, 85–31, 85–52 and 85–53) had apparently normal leaf morphology and did produce tubers (Fig. 4). Amongst the ten selected transgenic events, we found that the transgenic event 85–31 was consistently shorter (Fig. S3).

## Discussion

In this study, we demonstrate, for the first time, the production of a Brassicales-specific glucosinolate defense compound, BGLS, in potato. The BGLS pathway from *A. thaliana* was transferred to potato using two multigene constructs – 35Sprom::ORF2 and rbcS1prom::ORF1-GGP1—each containing a polycistronic transgene encoding three coding sequences. The stable genomic integration was done in two consecutive transformations similar to what was done previously in tobacco (Møldrup et al. 2012). The conversion of the intermediate PATH into BGLS in the untransformed potato evidenced the presence of glucosyltransferase and sulfotransferase activity in the green areas of potato plants, as also observed in the taxonomically related tobacco (Geu-Flores et al. 2009a, b).

Transformation of the selected transgenic event with the second polycistronic construct encoding the first three genes of the pathway (CYP79A2, CYP83B1 and GGP1) gave rise to BGLS-producing potato transgenic events. A much lower level of BGLS was observed in the transgenic potatoes (1.91 to 5.18 pmol/mg) in comparison with the levels reported in BGLS-producing tobacco (0.3 to 0.5 nmol/mg, Møldrup et al. 2012). Moreover, there was no apparent correlation between insertion copy number and BGLS production. One factor that could explain at least part of the difference in production level is that, in tobacco, all genes were driven by the high-expressing constitutive 35S CaMV promoter, whereas in potato the first three biosynthetic genes (CYP79A2, CYP83B1 and GGP1) were driven by the leaf-specific rubisco promoter and only the last three genes by the 35S CaMV promoter. The rubisco promoter has been much less studied and may not be as high as when using the 35S CaMV promoter. The rationale for using the rubisco promoter was to avoid the production of BGLS in the tubers as this could alter the taste and, thus, not be considered appropriate from a consumer perspective.

The total number of transgenic events screened was 27 and 50 in first and second transformation respectively. Out of the 50 transgenic events that were genotyped to contain all six genes and analyzed for BGLS accumulation, 29 transgenic events presented undetectable levels of BGLS in the plant tissues and of the remaining 21, only one transgenic event presented a higher BGLS. In our experience, the number of transgenic events is enough to test the utility of a transgene in potato, however, it might have been too low in the case of the introduction of the two transgenes to identify a transgenic event with high expression. In an experiment to study the expression of the reporter gene GUS in Arabidopsis, approximately 80% of low-expressors and a bit less than 20% of high-expressors were observed (Butaye et al. 2005). Therefore, it is possible that the number of transgenic events analyzed was not large enough to identify high BGLS accumulators.

Transgenic potato plants with BGLS production displayed morphological differences compared to the untransformed potato — smaller plant height, abnormally thick and curlier leaves – and only five out of ten transgenic events had normal growth and produced tubers. This represents 50% of abnormal phenotypes of the ORF2/ORF1-GGP1 transgenic events. Abnormalities related to the transformation process have been reported to be genotype-dependent and ranging from 1 to 21% (Heeres et al. 2002). Our experience with Désirée is on the low frequency range of 1 to 5% (data not shown). Therefore, this negative impact on growth must be related to the BGLS biosynthetic pathway either directly through any intermediates or through production of phytotoxic byproducts in potato. However, the end product, BGLS, does not seem to be responsible for this abnormal phenotype because the transgenic events with highest BGLS accumulation had a normal phenotype. The compartmentation of the biosynthesis and degradation of glucosinolates is essential to prevent the production of biologically active compounds (Halkier et al. 2006). In this study and in the similar study in tobacco, the BGLS pathway is targeted to the cytosol as in the native *A. thaliana.* It is an open question why the lower-producing transgenic potato show an abnormal phenotypes and the higher-producing tobacco does not. The presence of myrosinase activity in the cytosol of potato cells could lead to a continuous degradation of glucosinolates into phytotoxic products resulting in the abnormal growth. This aspect should be further investigated. For example, could metabolite profiling of the ORF2/ORF1-GGP1 transgenic potato help identifying products from glucosinolates degradation and other byproducts?

New gene constructs using other promoters and or attaching signal sequences to target the enzymes to other subcellular compartments such as chloroplasts, endoplasmic reticulum, vacuoles, or apoplast, could be tested to reduce the impact of the BGLS pathway on potato growth and tuber production. *ORF1-GGP1* transgene could be tested using another light-inducible promoter such as the *StLhca3* characterized recently as stronger than 35S promoter in potato (Miroshnichenko et al. 2020). It could also be tested using a wound-inducible promoter, but it would restrict the glucosinolate-mediated defense against insect herbivores. The use of weak and strong promoters driving both transgenes could provide insights into whether *ORF2* or *ORF1-GGP1* are responsible for the observed abnormal phenotypes.

In preliminary studies conducted to determine activity against two important pests of the potato: *Phytophthora infestans*, causing the late blight disease and *Premnotrypes suturicallus*, referred to as the Andean potato weevil (Alcazar et al. 1997), we observed a short term inhibition of *P. infestans* growth and a reduced egg oviposition and survival of the Andean potato weevil feeding on BGLS transgenic potato leaves. Further studies are needed to substantiate these preliminary results.

In conclusion, bioengineering of BGLS production into potato is a first step towards engineering the two-component defense system, the ‘mustard oil bomb’ characteristic of the plants in the Brassicales order, into potato. Next step in establishing the ‘mustard oil bomb’ is to introduce the glucosinolate-hydrolyzing enzyme, the myrosinase, in a separate space from the cytosol-localized biosynthetic pathway e.g. the apoplast, to enable hydrolysis of BGLS to the toxic benzylisothiocyanate upon attack. Bioengineering of the glucosinolate-myrosinase system in potato is expected to provide transgenic plants with a novel defense mechanism to reduce the impact of pests and diseases. Future investigations will aim at measuring Andean potato weevil efficacy and field performance of such transgenic potato plants. Potential benefit for farmers is a better quality of tubers and lower use of toxic synthetic chemicals.

## Materials and methods

### Gene constructs

The coding sequences of six BGLS biosynthetic genes were assembled into two plasmids: 35Sprom::ORF2 and rbcS1prom:ORF1-GGP1, each of which contains a single polycistronic open reading frame consisting of three *Arabidopsis thaliana* coding sequences separated by 2A sequences made of 18 amino acids (de Felipe et al. 2006) (Fig. 1). The previously reported 35Sprom::ORF2 plasmid consists of the coding sequences of the sulfotransferase (*SOT16,* GenBank # 843,750), glucosyltransferase (*UGT74B1,* GenBank # 839,022) and *C-S* lyase (*SUR1,* GenBank # 816,585) genes driven by the CaMV 35S promoter within the backbone of the pCAMBIA 2300 vector (Geu-Flores et al. 2009a). The order of the coding sequence followed the same order as previously (Geu-Flores et al. 2009a) and with *SUR1* placed last in the construct as the corresponding protein is not stable when tagged (Mikkelsen et al. 2004). The vector backbone bears the *nptII* gene driven by the CaMV 35S promoter for transgenic event selection. We assembled the rbcS1prom:ORF1-GGP1 plasmid containing the coding sequences of two cytochromes P450 (*CYP79A2*, GenBank # 830,408 and *CYP83B1*, GenBank # 829,277) genes which were referred to as ORF1 in the tobacco work (Geu-Flores et al. 2009b) and the γ-glutamyl peptidase 1 (*GGP1*, GenBank # 829,176) gene driven by the promoter of the rubisco small subunit (*rbcS1*) gene of chrysanthemum (*Chrysanthemum morifolium* Ramat.). To build this construct, a PCR fragment containing *CYP83B1, CYP79A2* and *GGP1* was amplified from the previously generated 35Sprom::ORF1-GGP1 plasmid (Møldrup et al. 2012) using primers 5′-GGCTTAAUACTAGTTTAGGTTGGATACACATGTG-3´ and 5′-GGTTTAAUACTAGTATGGTGGAGCAAAAGAGAT-3´. The PCR fragment was cloned into the USER-compatible vector pCAMBIA3300-35S (Nour-Eldin et al. 2006), which provides the *bar* gene driven by the CaMV 35S promoter for transgenic event selection.

### Plant transformation and selection of transgenic events

The individual plasmids 35Sprom::ORF2 and rbcS1prom:ORF1-GGP1 were transferred separately into *Rhizobacterium radiobacter* (syn. *Agrobacterium tumefaciens*) strain EHA105. Potato plants of the variety Désirée (CIP genebank 800,048, referred also to as near-isogenic control (NIC) were grown in vitro in a growth room at 14–16 °C, 70% relative humidity and 16 h photoperiod (2,000 lx). These plants were transformed sequentially with the transgenes *ORF2* and *ORF1-GGP1* using a modified version of a previously described protocol (Cuellar et al. 2006) as follows. Colonies of *R. radiobacter* (syn. *A. tumefaciens*) harbouring the 35Sprom::ORF2 plasmid were taken with a scalpel that was subsequently used to cut the petiole of four-week-old excised leaves longitudinally. Infected explants were placed in solid Murashige-Skoog (MS) medium supplemented with 2% sucrose and incubated for 24 h at 18 ± 2 °C under artificial light (16 h light/8 h dark). The infected explants were then transferred to selective regeneration medium (4.3 g/L MS salts, 20 g/L sucrose, 3 g/L Phytagel™, 0.02 mg/l gibberelic acid (GA_3_), 0.02 mg/L naphthalene acetic acid (NAA) and 2 mg/L zeatin riboside) supplemented with 200 mg/L carbenicillin (to eliminate residual *R. radiobacter* (syn. *A. tumefaciens*) bacteria) and 100 mg/L kanamycin (to select for the transgenic events). Three-week-old shoots were transferred to tubes containing MSA medium (MS salts, 0.4 mg/L thiamin, 2 mg/L glycine, 0.5 mg/L nicotinic acid, 0.5 mg/L pyridoxine, 0.1 mg/L gibberellic acid, 2% sucrose and 3 g/L Phytagel™) supplemented with 100 mg/L carbenicillin. Identification of transgenic events was done by genotyping ORF2 putative transgenic plants by PCR amplification of a 661-bp fragment from *ST* using primers 5′-CACTCCTCAAACGAAACCCTCA-3´ and 5′-TCCTCCACTAAGCCATCAATACGA-3´. One selected transgenic event obtained from this first transformation was later transformed with the rbcS1prom:ORF1-GGP1 plasmid following the method described above. Regenerants were grown under selection on a solid regeneration medium supplemented with 250 mg/L carbenicillin and 2 mg/L phosphinothricin. The identification of the transgenic events with the second T-DNA was done by PCR amplification of a 661-bp fragment from *ST* (see above) and a 515-bp fragment from *CYP79A2* obtained with primers 5′-GATGAGGAGAGTGGTGGCA-3´ and 5′-TAGGCTTCCCGTCAGTGT-3´.

### Southern blotting

DNA from transgenic potato plants carrying *ORF2* transgene was extracted according to previously described methods (Cuellar et al. 2006). Twenty μg of total DNA was digested with the restriction enzyme *Xba*I to analyze for transgenic events carrying the *ORF2* transgene. This restriction enzyme was chosen because it is located at 4610 bp from the left border sequence which produces a DNA fragment containing the *nptII* gene and potato genomic DNA ending by a *Xba*I site. Hence, one or more fragments of different sizes depending where the plant *Xba*I site is positioned is expected for each transgenic event depending on how many insertions of the T-DNA have occurred. Digested DNA was separated in 0.8% agarose gels and transferred to positively charged nylon membranes (Hybond-N^+^, Amersham). Radioactively labelled DNA fragments of 661 bp and 597 bp, obtained by PCR amplification of a region of the *SOT16* and *nptII* coding sequences from the 35Sprom::ORF2 plasmid, were used as probes to analyze transgene insertion number. Membrane hybridizations followed standard procedures. Hybridization results were visualized by autoradiography using Kodak X-ray films exposed for 24 h at − 70 °C.

For the analyses of transgenic events containing both the *ORF2* and *ORF1-GGP1* transgenes, approximately 20 µg DNA was digested with *Eco*RI, electrophoresed in 0.8% agarose and blotted onto a nylon membrane as described above. The probe was a digoxigenin (DIG)-labelled 269-bp *bar* fragment obtained by PCR amplification using the rbcS1prom:ORF1-GGP1 plasmid as a template, primers 5′-ACCATCGTCAACCACTACAT-3′ and 5′-TTCAGCAGGTGGGTGTAGAG-3′ and DIG-labelled dNTPs (Roche). Pre-hybridization (1 h) and hybridization (16 h) were performed at 68 °C in standard hybridization buffer (Roche). Labelling and detection with the non-radioactive method was done according to the manufacturer’s protocols (Roche).

### Quantitative real-time PCR analysis

Total RNA was isolated from leaves of transgenic plants grown in vitro using TRIzol as indicated by the manufacturer (Invitrogen). The first-strand cDNA synthesis was performed using the SuperScript® III First-Strand Synthesis System (Invitrogen). The cDNA products were used as templates in real time quantitative PCR (RT-qPCR). *ORF2* transgene expression was quantified by RT-qPCR using primers 5′-TCCTGGCTTCCCTCACTACGA-3′ and 5′-TCTCGTCTGCTGCAATGGCTTCG-3′ in the *SUR1* coding sequence in a 20 μL reaction mixture consisting of 10 μL iQ SYBR Green Supermix, 0.25 μM of each primer and 1 μL of cDNA (20 ng/μL). The PCR cycling program was as follows: 95 °C for 2 min, 40 cycles of 94 °C for 15 s, 55 °C for 20 s, 72 °C for 20 s, 82 °C for 1 s and one cycle of 72 °C for 10 min, followed by a melting curve program from 65 to 98 °C with a heating rate of 0.1 °C per second and a cooling step of 10 °C. Each PCR was repeated three times as technical replicates. The potato mitochondrial cytochrome oxidase (*cox1*) gene (GenBank X83206.1) was used as a reference gene (Weller et al. 2000) and its amplification was achieved using primers 5′-CGTCGCATTCCAGATTATCCA-3′ and 5′-CAACTACGGATATATAAGAGCCAAAACTG-3′. The melting curve analysis gave a melting point of 82.4 °C and 79 °C for the *ORF2* transgene and the *cox1* gene, respectively and evidenced a single PCR product in both cases. The level of expression of *ORF2* transgene was analyzed with REST software.

### Analysis of BGLS levels in transgenic plants carrying both ORF2 and ORF1-GGP1 transgenes

200 mg of five-week-old leaves from transgenic in vitro plantlets were homogenized in 400 µL of 85% methanol containing 0.02 mM sinigrin (SigmaAldrich) as an internal standard. We used the commercially available glucosinolate sinigrin as internal standard. Untransformed plants (NIC) were used as a control. Glucosinolates were measured using the desulfoglucosinolate method as described previously (Geu-Flores et al. 2009a; Hansen et al. 2007). Six biological replicates (n = 6) were used in the in vivo feeding of PATH using leaves from 3–4-week-old in vitro grown ORF2 transgenic plants as well as in the ORF2/ORF1-GGP1 transgenic plants. For quantification of BGLS, we used the response factor previously described by Brown et al. (2003). Difference in BGLS production between NIC and transgenic plants were analyzed using Dunnett's test at a significance level of *P* < 0.05.

BGLS-producing transgenic events were grown in the greenhouse under natural light and temperature. Observations of plant height were done at 4, 10, 16 and 18 weeks after planting while harvest was done after plant senescence 18 weeks after planting. We used two repetitions per transgenic event.

## Supplementary Information

Below is the link to the electronic supplementary material.

Supplementary file1 (DOCX 192 kb)

## Abbreviations

BGLS: Benzylglucosinolate
rbcS1prom: Promoter of the ribulose-1,5 bisphosphate carboxylase small subunit gene family of chrysanthemum (*Chrysanthemum morifolium* Ramat.)
MeOH: Methanol
ISTD: Internal standard
