# Correction to: Bioengineering potato plants to produce benzylglucosinolate for improved broad-spectrum pest and disease resistance

**DOI:** 10.1007/s11248-021-00265-8

**Published:** 2021-06-14

**Authors:** M. E. González-Romero, C. Rivera, K. Cancino, F. Geu-Flores, E. G. Cosio, M. Ghislain, B. A. Halkier

**Affiliations:** 1 Applied Biotechnology Laboratory, International Potato Centre, P.O. Box 1558, Lima, 12 Peru; 2 Department of Plant and Environmental Sciences, DynaMo Center, University of Copenhagen, Thorvaldsensvej 40, 1871 Frederiksberg, Denmark; 3 Department of Plant and Environmental Sciences, Copenhagen Plant Science Center & Section for Plant Biochemistry, University of Copenhagen, Thorvaldsensvej 40, Frederiksberg, Denmark; 4 Chemistry Section, Pontificia Universidad Católica del Perú, Av. Universitaria 1801, Lima, 15088 Peru; 5 Present Address: Universidad Nacional Agraria La Molina, Av. La Molina s/n, Lima, 12 Peru; 6 Present Address: Pathology Department, Instituto Nacional de Enfermedades Neoplásicas, Av. Angamos Este 2520, Lima, 15038 Peru

## Correction to: Transgenic Res 10.1007/s11248-021-00255-w

In the above mentioned publication, a third affiliation was erroneously indicated for M. E. González-Romero where it should have been two. The original article has been corrected and the correct affiliations are also published here.

The article Bioengineering potato plants to produce benzylglucosinolate for improved broad-spectrum pest and disease resistance, written by M. E. González-Romero, C. Rivera, K. Cancino, F. Geu-Flores, E. G. Cosio, M. Ghislain and B. A. Halkier, was originally published electronically on the publisher’s internet portal on 06 May 2021 without open access. With the author(s)’ decision to opt for Open Choice the copyright of the article changed on 19 May 2021 to © The Author(s) 2021 and the article is forthwith distributed under a Creative Commons Attribution 4.0 International License, which permits use, sharing, adaptation, distribution and reproduction in any medium or format, as long as you give appropriate credit to the original author(s) and the source, provide a link to the Creative Commons licence, and indicate if changes were made.

The images or other third party material in this article are included in the article’s Creative Commons licence, unless indicated otherwise in a credit line to the material. If material is not included in the article’s Creative Commons licence and your intended use is not permitted by statutory regulation or exceeds the permitted use, you will need to obtain permission directly from the copyright holder.

To view a copy of this licence, visit http://creativecommons.org/licenses/by/4.0/.

