# Supplementary material for: Bioengineering potato plants to produce benzylglucosinolate for improved broad-spectrum pest and disease resistance
Source: Transgenic Res. 2021 May 6;30(5):649–60. doi: 10.1007/s11248-021-00255-w (PMC8478770; doi:10.1007/s11248-021-00255-w)
Supplement: Supplementary file 1 — Supplementary file1 (DOCX 192 kb) [file 11248_2021_255_MOESM1_ESM.docx]

**SUPPLEMENTARY INFORMATION**

**SUPPLEMENTARY FIGURES**

**Fig. S1.** Analysis of *ORF2* transgene expression in 27 transgenic events. The expression level is shown relative to transgenic event 112 that was taken as the reference due to its lowest expression value. Error bars represent standard deviation, n=5.

**A B**

**M NIC Event P M NIC Transgenic events P**

**
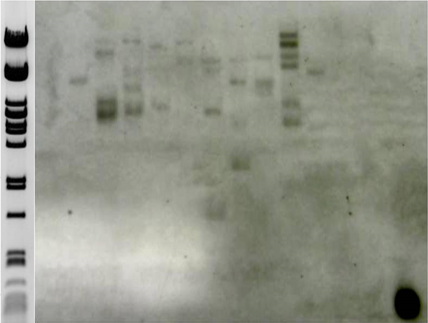

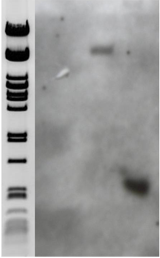
 85 26 23 24 31 16 52 49 4 11 53**

5077

11490

5077

11490

2838

4507

4507

2838

1159

1159

597

597

269

269

**Fig. S2.** Southern blotting analysis of transgenic potato events. A. Transgenic event 85 bearing the *ORF2* transgene; and B. Ten selected transgenic events bearing both the *ORF2* and *ORF1-GGP1* transgenes were analyzed for insertion number. Hybridization was done using digoxigenin-labelled probes of 597 bp of *nptII* gene and 269 bp of *bar* gene, respectively. Other lanes are as follows: M is molecular marker weights (*Pst*I fragments of λ DNA; NIC is the untransformed potato variety Désirée; and P is the probe itself.

**Fig. S3.** Growth of ten BGLS-producing transgenic events in the greenhouse which have the *ORF2* and *ORF1-GPP1* transgenes, the transgenic event 85 which has the *ORF2* transgene, and the untransformed Désirée as near-isogenic line (NIC).
